# Increased use of malaria rapid diagnostic tests improves targeting of anti-malarial treatment in rural Tanzania: implications for nationwide rollout of malaria rapid diagnostic tests

**DOI:** 10.1186/1475-2875-11-221

**Published:** 2012-07-02

**Authors:** Irene M Masanja, Majige Selemani, Baraka Amuri, Dan Kajungu, Rashid Khatib, S Patrick Kachur, Jacek Skarbinski

**Affiliations:** 1 INESS programme, Ifakara Health Institute, PO Box 78373, Dar es Salaam, Tanzania; 2 INDEPTH Network Effectiveness and Safety Studies of Antimalarial Drugs in Africa, Accra, Ghana; 3 Swiss Tropical and Public Health Institute, Socinstrasse 57, Basel, CH-4002, Switzerland; 4 Universität Basel, Petersplatz 1, Basel, CH-4003, Switzerland; 5 Malaria Branch, Centers for Disease Control and Prevention, Atlanta, GA, USA

**Keywords:** Malaria rapid diagnostic tests, ACT, HDSS, INDEPTH network, Tanzania

## Abstract

**Background:**

The World Health Organization recommends parasitological confirmation of all malaria cases. Tanzania is implementing a phased rollout of malaria rapid diagnostic tests (RDTs) for routine use in all levels of care as one strategy to increase parasitological confirmation of malaria diagnosis. This study was carried out to evaluated artemisinin combination therapy (ACT) prescribing patterns in febrile patients with and without uncomplicated malaria in one pre-RDT implementation and one post-RDT implementation area.

**Methods:**

A cross-sectional health facility surveys was conducted during high and low malaria transmission seasons in 2010 in both areas. Clinical information and a reference blood film on all patients presenting for an initial illness consultation were collected. Malaria was defined as a history of fever in the past 48 h and microscopically confirmed parasitaemia. Routine diagnostic testing was defined as RDT or microscopy ordered by the health worker and performed at the health facility as part of the health worker-patient consultation. Correct diagnostic testing was defined as febrile patient tested with RDT or microscopy. Over-testing was defined as a non-febrile patient tested with RDT or microscopy. Correct treatment was defined as patient with malaria prescribed ACT. Over-treatment was defined as patient without malaria prescribed ACT.

**Results:**

A total of 1,247 febrile patients (627 from pre-implementation area and 620 from post-implementation area) were included in the analysis. In the post-RDT implementation area, 80.9% (95% CI, 68.2-89.3) of patients with malaria received recommended treatment with ACT compared to 70.3% (95% CI, 54.7-82.2) of patients in the pre-RDT implementation area. Correct treatment was significantly higher in the post-implementation area during high transmission season (85.9% (95%CI, 72.0-93.6) compared to 58.3% (95%CI, 39.4-75.1) in pre-implementation area (p = 0.01). Over-treatment with ACT of patients without malaria was less common in the post-RDT implementation area (20.9%; 95% CI, 14.7-28.8) compared to the pre-RDT implementation area (45.8%; 95% CI, 37.2-54.6) (p < 0.01) in high transmission. The odds of overtreatment was significantly lower in post- RDT area (adjusted Odds Ratio (OR: 95%CI) 0.57(0.36-0.89); and much higher with clinical diagnosis adjusted OR (95%CI) 2.24(1.37-3.67)

**Conclusion:**

Implementation of RDTs increased use of RDTs for parasitological confirmation and reduced over-treatment with ACT during high malaria transmission season in one area in Tanzania. Continued monitoring of the national RDT rollout will be needed to assess whether these changes in case management practices will be replicated in other areas and sustained over time. Additional measures (such as refresher trainings, closer supervisions, etc.) may be needed to improve ACT targeting during low transmission seasons.

## Background

The World Health Organization (WHO) recommends that clinically suspected malaria be confirmed parasitologically prior to treatment [1]. Diagnosis based on signs and symptoms has varied sensitivity and specificity depending on clinical features present, age, and transmission-associated acquired immunity [2]. Microscopic analysis of blood films for malaria parasites has been the most commonly used method for parasitological confirmation of malaria infection. However, due to reports of poor quality malaria microscopy [3,4] and the logistical, personnel and financial resources required to increase coverage of quality microscopy services, many endemic countries in sub-Saharan Africa have opted to use malaria rapid diagnostic tests (RDTs) to expand the use of parasitological-based malaria diagnosis [5,6].

Tanzania mainland introduced the use of artemether-lumefantrine (AL), an artemisinin-based combination therapy (ACT), as a first line treatment for uncomplicated malaria in 2006 [7]. In early 2009, a phased rollout of RDTs was initiated in all levels of care to complement microscopy services for parasitological confirmation of malaria prior to treatment (National guideline for the use of rapid malaria diagnostic tests in Tanzania, 2007, National Malaria Control Programme, Dar es Salaam, Tanzania: unpublished). This roll-out is expected to cover the entire country before end of year 2012. Since the adoption of RDT policy and increased rollout of this strategy in sub-Saharan Africa, there has been limited evidence of the performance of RDTs in routine use and their impact on case management practices, on a wider scale, outside research settings.

Using a pair of cross-sectional health facility surveys conducted in two areas of Tanzania in 2010, this study assessed the impact of RDTs by comparing malaria case management practices in a post-RDT implementation area with a pre-RDT implementation area. The assessment aimed on determining ACT targeting accuracy, i.e. the proportion of patients presenting to ACT provider for initial illness consultation with fever or history of fever in the last 48 h who were prescribed AL; and provider compliance, i.e. the proportion of patients presenting to an ACT provider for initial illness consultation with fever or history of fever in the last 48 h and blood slide confirmed malaria parasitaemia, who were prescribed AL.

In the post-RDT implementation area, Rufiji Health and Demographic Surveillance System (HDSS) located in Rufiji District, RDTs were introduced for routine use in November 2009 as part of the national rollout. In the pre-RDT implementation area, Ifakara HDSS located in Kilombero and Ulanga Districts, RDT were not introduced as part of the national roll-out, but were available in seven health facilities (out of 79 health facilities in Kilombero and Ulanga districts) as part of small scale research study; the ACCESS programme, since 2008 [8].

## Methods

INDEPTH Network Effectiveness and Safety Studies of Antimalarial Drugs in Africa platform (INESS) operates in HDSSs in Rufiji District, Coast Region, and in Kilombero and Ulanga Districts, around the town of Ifakara, Morogoro Region, Tanzania [9]. The platform assesses safety and community effectiveness of anti-malarial drugs in real life health systems. As part of this effort, INESS conducts assessments of the quality of malaria case management using a series of health facility surveys to evaluate ACT prescribing patterns among febrile patients with and without parasitological confirmed uncomplicated malaria.

### Study area

The study was conducted in health facilities located in the Rufiji and Ifakara HDSS areas, in March and October 2010 corresponding to high and low malaria transmission seasons respectively at those sites. The Rufiji HDSS has been operational since 1998 and contains a population of approximately 85,000 people [10]. Malaria rapid tests were available on a limited scale as part of demonstration project in 12 health facilities in the Rufiji HDSS since 2007 [11] and in all facilities after the national RDT rollout in 2009. Ifakara HDSS has been operational since 1996 and contains a population of approximately 99,000 people [12]. Early in 2008, the ACCESS project introduced RDT in seven (out of 79) health facilities in Kilombero and Ulanga districts; four out of these seven facilities are within the HDSS area [8]. Although use of RDTs under ACCESS project was under research settings, health workers received training and guidelines similar to those provided in the rollout areas for routine case management. These were modified versions of the generic guides provided by the WHO. The two areas differ in terms of support supervision and stock supply for RDTs. In Rufiji, supervision and acquisition of supplies was to follow routine practices in existing health system; those under ACCESS project were assessed by project investigators who were also responsible for replenishing facility supplies of RDTs.

### Health facility surveys

Cross-sectional health facility cluster surveys were conducted in March and October 2010. A cluster was defined as all outpatient consultations in a health facility conducted in one day during regular working hours. All government and non-government health facilities that provide outpatient care to the HDSS population were included (16 in Rufiji HDSS and 14 in Ifakara HDSS). Each facility was visited for two to three days with the goal to collect data on 720 patients per HDSS per year in order to estimate the proportion of patients with uncomplicated malaria correctly treated with ACT with 10% confidence, assuming 20% of all patients present with uncomplicated malaria, 75% of patients with uncomplicated malaria are treated with ACT, and a design effect of 2 for cluster sampling

All outpatients presenting for initial illness consultation on a day of a survey, and who consented to participate in the survey, were interviewed prior to leaving the health facility. Using standardized questionnaires developed in English and translated into Kiswahili, information on history of fever, health worker’s diagnoses, laboratory tests, medications prescribed and counselling messages were collected. In addition, a reference blood film for malaria microscopy was collected on every patient. Patients with severe malaria were excluded from the survey. Moreover, interviews were carried out with health workers providing outpatient consultations and collected information on pre-service training, work experience, in-service training and receipt of supervision. Assessment of the level of staffing, availability of diagnostics, medications and other medical supplies was done at the health facility.

### Laboratory procedure

Blood films were stained with 10% Giemsa and thick and thin films were examined by study microscopists at centralized locations in Rufiji and Ifakara HDSS sites. A second reading was conducted by a reference laboratory technician at the Ifakara Health Institute Bagamoyo Research and Training Unit. Parasite densities were calculated by counting the number of asexual stage parasites per 200 white blood cells (WBCs) and assuming an average of 8,000 WBC per microlitre of blood. A blood film was considered negative if no parasites were found after counting 100x high power fields. Blood film results were made available to the respective health facilities between five to seven days after the day of survey.

### Definitions

National malaria treatment guidelines and clinical information from the exit interview were used to determine when diagnostic testing was indicated, and the same plus study blood film results to determine when prescription of ACT was indicated. In the treatment guidelines, malaria diagnostic testing was indicated for patients with febrile illness (history of fever in the last 48 h), and this was assessed through exit interviews. Malaria was defined as a patient with febrile illness as determined by exit interview, and reference blood film positive for malaria parasites. Routine diagnostic testing was defined as RDT or microscopy ordered by the health worker and performed at the health facility as part of the health worker-patient consultation. Correct diagnostic testing was defined as febrile patient tested with RDT or microscopy. Over-testing was defined as a non-febrile patient tested with RDT or microscopy. Correct treatment was defined as patient with malaria prescribed ACT. Over-treatment was defined as patient without malaria prescribed ACT. ACT stock out referred to absence of all types of AL blister packs.

### Data management and analysis

Data were double entered in EPIDATA (version 3.1, EPIDATA Association, Odense, Denmark) and analysed using STATA (version 11.0, STATA Corporation, College Station, USA) using survey procedures that account for clustering. An alpha level of 0.05 was used for all significance tests. Pregnant women (N = 60) were excluded from the analysis as quinine rather than ACT is the recommended treatment of malaria in the first trimester. Descriptive analysis was performed to derive proportions for different outcome measures. Logistic regression was done to identify the impact of diagnostics on prescription of AL and overtreatment to patients receiving AL.

### Ethical clearance

All components of the INESS platform were reviewed by the Tanzanian National Institutes of Medical Research and IHI’s Ethical Review Boards (IHI/IRB/No.A67-2009), Dar es Salaam, Tanzania.

## Results

A total of 1,531 patients were interviewed (742 in Rufiji HDSS and 789 in Ifakara HDSS) and 1,471 non-pregnant patients with complete data were included in the analysis. Almost half of all patients in both areas were children age <5 years (Table 1). Use of insecticide-treated bed nets, availability of ACT and diagnostic testing (RDT and microscopy) differed significantly between the two areas. In particular, more patients in Rufiji HDSS (post-RDT implementation area) were seen in a health facility with ACT in stock (93.8%) or with any diagnostic capacity (RDT or microscopy) (74.2%) compared to the Ifakara HDSS (pre-RDT implementation area) where, 42.8% were seen in a health facility with ACT in stock and 32.6% seen in a health facility with diagnostic capacity. Moreover, the prevalence of uncomplicated malaria varied significantly between Rufiji HDSS (high season 19.2% and low season 7.2%) and Ifakara HDSS (high season 9.4% and low season 4.2%), respectively.

**Table 1:** Characteristics of patients in health facility surveys conducted in pre- and post-RDT implementation HDSS areas

**Characteristic**	**Ifakara HDSS (pre-RDT implementation) (N = 761)**		**Rufiji HDSS (post-RDT implementation) (N = 710)**		**P-value**
	**n/N**	**% (95%CI)**	**n/N**	**%(95%CI)**	
Female	441/761	58.0 (54.7-61.1)	347/710	48.9 (44.9-52.9)	<0.01
Child aged <5 years	352/761	46.3 (41.8-50.8)	349/710	49.2 (44.4-53.9)	0.56
Used insecticide-treated bed net previous night	575/761	75.6 (71.3-79.4)	344/710	48.5 (40.9-56.1)	< 0.01
Used anti-malarial prior to health facility visit	37/761	4.9 (3.2-7.3)	20/710	2.8 (1.5-5.3)	0.14
Seen in high transmission season (March 2010)	384/761	50.5 (40.2-60.7)	375/710	52.8 (39.7-65.5)	0.78
Seen in low transmission season (October 2010)	377/761	49.5 (39.3-59.8)	335/710	47.2 (34.5-60.3)	
Seen in HF with ACT in stock	326/761	42.8 (30.9-55.7)	666/710	93.8 (86.9-97.2)	0.01
Seen in HF with RDT or BS in stock	248/761	32.6 (21.4-46.1)	527/710	74.2 (61.1-84.1)	<0.01
Seen in HF with a RDT in stock	132/761	17.4 (10.6-27.6)	343/710	48.3 (35.2-61.7)	<0.01
Seen in a HF with BS in stock	242/761	31.8 (22.0-43.5)	397/710	55.9 (41.9-69.1)	0.28
Fever prevalence	627/761	82.4 (78.3-85.8)	620/710	87.3 (83.3-90.5)	0.06
Uncomplicated malaria prevalence	54/761	7.1 (5.1-9.8)	96/710	13.5 (10.3-17.6)	<0.01
High transmission season	36/384	9.4 (6.2-13.9)	72/375	19.2 (14.3-25.3)	<0.01
Low transmission season	16/377	4.2 (2.5-7.2)	24/335	7.2 (4.2-12.0)	0.21

Results in Table 2 describe the use of RDTs between the two areas. More patients in the post- RDT area received a diagnostic test 62.1% (95%CI: 50.3- 72.6) as compared to a pre-RDT area 46.5% (95%CI: 36.3- 57.1) (p = 0.05). Use of RDTs was significantly higher in post-RDT area whereas microscopy use was more common in the pre-RDT area. Overall correct testing and over-testing did not differ significantly between two areas.

**Table 2:** Use of diagnostics to patients with and without fever in pre- and post-RDT implementation HDSS areas

**Characteristic**	**Ifakara HDSS (pre-RDT implementation area)**		**Rufiji HDSS (post-RDT implementation area)**		**P-value (site)**
	**n/N**	**%(95%CI)**	**n/N**	**%(95%CI)**	
All patients tested by either RDT or microscopy	354/761	46.5 (36.3-57.1)	441/710	62.1 (50.3-72.6)	0.05
RDT	132/761	17.4 (11.0-26.2)	309/710	43.5 (32.4-55.4)	< 0.01
Microscopy	242/761	31.8 (28.5-35.2)	132/710	18.6 (15.8-21.7)	0.05
Correct testing: Patients with fever tested either RDT or microscopy	314/627	50.1 (38.9-61.2)	400/620	64.5 (51.8-75.5)	0.09
RDT	123/627	19.6 (12.4-29.6)	282/620	45.5 (33.3-58.2)	< 0.01
Microscopy	210/627	33.5 (29.8-37.3)	118/620	19.0 (16.0-22.3)	0.04
Over-testing: Patients without fever tested with either RDT or microscopy	40/134	29.9 (16.5-47.9)	41/90	45.6 (32.9-58.8)	0.15
High season	5/60	8.3 (3.6-18.0)	26/56	46.4 (33.2-60.2)	<0.01
Low season	35/74	47.3 (24.0-71.9)	15/34	44.1 (22.0-68.9)	0.86

Table 3 shows ACT prescriptions to malaria patients as categorized by use of diagnostic tests (RDT and microscopy) or clinically. In both areas, most RDT-positive patients received ACT, but only about half microscopy-positive patients received ACT. RDT-negative patients were significantly less likely to be treated with ACT in the post-RDT implementation area than the pre-RDT implementation area. The proportion of patients receiving ACT based on clinical diagnosis alone did not differ between the two sites. In the multivariate analysis (Table 4), the odds of febrile patients receiving ACT were highest with clinical diagnosis (adjusted OR 95%CI: 2.24(1.37-3.67).

**Table 3:** ACT† prescription according to tests results, and clinical malaria* in pre- and post-RDT implementation areas

	**Ifakara HDSS (pre- RDT implementation)**		**Rufiji HDSS (post- RDT implementation)**		**P-value**
	**n/N**	**%(95% CI)**	**n/N**	**%(95% CI)**	
RDT positive	22/30	73.3 (53.1-87.0)	79/95	83.2 (69.2-91.6)	0.32
RDT negative	28/104	26.9 (16.4-40.9)	17/217	7.8 (4.7-12.7)	<0.01
Microscopy positive	62/110	56.4 (41.5-70.1)	15/36	41.7 (22.1-64.3)	0.28
Microscopy negative	7/135	5.2 (2.1-12.1)	5/96	5.2 (2.3-11.6)	0.99
Clinical diagnosis of malaria and no diagnostic test performed	165/313	52.7 (42.9-62.4)	99/220	45.0 (34.3-56.2)	0.33
No clinical diagnosis of malaria and no diagnostic test performed	7/94	7.5 (2.9-15.8)	2/49	4.1 (1.1-14.4)	0.45

* Clinical diagnosis of malaria based on presence of fever in patients who did not receive a malaria diagnostic test **† =** Artemether lumefantrine.

**Table 4:** Multivariate analysis of ACT† prescription according to tests results, and clinical malaria* for all febrile patients in pre- and post-RDT implementation areas

**Variable**	**Crude Odd ratio**	**95%CI**	**p-value**	**Adjusted Odd ratio**	**95%CI**	**p-value**
**Type of diagnosis**						
mRDT	Ref					
Microscopy	0.59	0.37-0.93	0.02	0.61¥	0.38-1.01	0.054
Clinical diagnosis	1.29	0.87-1.91	0.21	1.30¥	0.87-1.95	0.201
**Season**						
High season	Ref					
Low season	0.64	0.43-0.97	0.036	0.67†	0.45-1.00	0.048
**Site**						
Ifakara HDSS (pre-mRDT)	Ref					
Rufiji HDSS (post-mRDT)	0.74	0.49-1.12	0.15	0.72‡	0.48-1.10	0.127

¥: adjusted for season and site.

†: adjusted for type of diagnosis and site.

‡: adjusted for type of diagnosis and season.

Overall ACT use and correct treatment of malaria were about similar in the two areas, except during high transmission season (Table 5) but over-treatment of non-malaria patients with ACT was significantly higher in the pre-RDT area (39.1%; 95%CI 31.0-47.8) compared to the post-RDT area (24.7%; 95%CI 18.4-32.2), adjusted OR(95%CI) 0.57 (0.36-0.89); Table 6. Seasonal differences were noted in malaria prevalence (Table 1), testing rate (Table 2), correct treatment and over-treatment (Table 5).

**Table 5:** Prescription of ACT for all patients in post- and pre-RDT implementation areas with ACT in stock at health facility

**Characteristic**	**Ifakara HDSS (pre-mRDT implementation)**		**Rufiji HDSS (post-mRDT implementation)**		**P-value (site)**
	**n/N**	**%(95% CI)**	**n/N**	**%(95% CI)**	
All patients treated with ACT (overall)	241/587	41.1 (33.4-49.2)	217/666	32.6 (26.5-39.4)	0.10
High transmission season (March 2010)	155/332	46.7 (38.9-54.6)	119/349	34.1 (27.9-40.9)	0.02
Low transmission season (October 2010)	86/255	33.7 (22.6-47.0)	98/317	30.9 (20.7-43.4)	0.74
Correct treatment: Patients with uncomplicated malaria treated with ACT (overall)	26/37	70.3 (54.7-82.2)	76/94	80.9 (68.2-89.3)	0.22
High transmission season (March 2010)	14/24	58.3 (39.4-75.1)	61/71	85.9 (72.0-93.6)	0.01
Low transmission season (October 2010)	12/13	92.3 (54.2-99.2)	15/23	65.2 (36.2-86.1)	0.11
Over treatment: Patients without uncomplicated malaria treated with ACT (overall)	215/550	39.1 (31.0-47.8)	141/572	24.7 (18.4-32.2)	0.01
High transmission season (March 2010)	141/308	45.8 (37.2-54.6)	58/278	20.9 (14.7-28.8)	<0.01
Low transmission season (October 2010)	74/242	30.6 (19.6-44.3)	83/294	28.2 (18.2-41.1)	0.78

**Table 6:** Multivariate description of ACT over-treatment for all patients in post- and pre-RDT implementation areas

**Variable**	**Crude Odd ratio**	**95%CI**	**p-value**	**Adjusted Odd ratio**	**95%CI**	**p-value**
**Type of diagnosis**						
mRDT	Ref					
Microscopy	1.20	0.72-1.98	0.48	1.05¥	0.60-1.84	0.856
Clinical diagnosis	2.54	1.56-4.12	0.00	2.24¥	1.37-3.67	0.001
**Season**						
High season	Ref					
Low season	0.89	0.57-1.38	0.587	0.82 †	0.54-1.23	0.328
**Site**						
Ifakara HDSS(pre-mRDT)	Ref					
Rufiji HDSS(post-mRDT)	0.50	0.32-0.8	0.004	0.57‡	0.36-0.89	0.014

¥: adjusted for season and site.

†: adjusted for type of diagnosis and site.

‡: adjusted for type of diagnosis and season.

## Discussion

Parasitological confirmation of all malaria diagnoses is recommended by WHO and there is increasing evidence supporting the use of RDTs for clinical management of fever and malaria cases [13-15]. Many of these findings are based on operational research that assessed performance and accuracy of RDT use in clinical care as part of a pilot or research projects. This study evaluated the implementation of RDTs for routine use in the Tanzanian health system, under “real world” conditions. Most importantly, in the post-RDT implementation area correct treatment of malaria remained high (80.9%) and over-treatment of non-malaria patients was low (24.7%). This suggests that the routine use of RDTs might improve malaria case management.

Tanzania began and phased introduction of RDTs for routine malaria case management in 2008 and plans to achieve nationwide coverage by 2011 [National guideline for the use of rapid malaria diagnostic tests in Tanzania, 2007, National Malaria Control Programme, Dar es Salaam, Tanzania: unpublished]. The scaling up of health interventions is a complex undertaking and simply increasing coverage might not translate into an impact on the larger population [16,17]. By comparing the post-RDT implementation area to the pre-RDT implementation area, the impact of RDT roll-out in malaria case management can be assessed. First, the post-RDT implementation area had much higher availability of malaria diagnostic testing capacity; 74% of patients were seen in a health facility that had either RDTs or microscopy available compared to 32.6% of patients seen on the pre-RDT implementation area. However, availability of diagnostics alone does not improve malaria case management. In particular, more patients were tested for malaria in the post-RDT implementation area (62.1%) than the pre-RDT implementation area (46.5%), and more patients were correctly tested.

Despite the improvement seen in proportion of febrile patients receiving a test, this achievement is far from optimal. The reason could be associated with the much too frequent stock out of medical products affecting the Tanzania health system, including timely in-availability of testing materials/devices. However, results show that over-testing in post implementation area is also high. Over-testing may be associated with wasted resources as patients who do not meet clinical criteria for malaria diagnostic testing are tested. Although we did not assess reasons for over-testing, one may think that the problem may be contributed by lower understanding of case selection for the test, probably resulting from lack of experience in using the tests, unclear guideline, supportive supervision not focusing on the topic or even patient pressure to get tested. Post-implementation care quality improvement efforts should focus on ensuring that only persons who have clinical signs and symptoms of malaria such as a history of fever are tested.

Another important consideration for RDT implementation is assuring the diagnostic test performance. In this study, sensitivity and specificity of RDTs in the post-implementation area was adequate (>85%; see Additional file 1). The continued monitoring of RDT performance post-implementation is critical as poor malaria microscopy performance has been documented throughout sub-Saharan Africa. For the time being, RDTs appear to have better sensitivity and specificity than routine microscopy and might be critical in improving the overall quality of malaria diagnostic capacity in routine settings.

In addition, this study suggests that adherence to RDT results is reasonably high with 83.2% of RDT-positive patients receiving ACT and only 7.8% of RDT-negative patients receiving ACT. Adherence to diagnostic test results is critical if the implementation of RDTs is expected to reduce over-treatment. Unlike previous studies, this study suggests that health workers do adhere to RDT results, even after routine implementation of RDTs. In addition, findings from this study also confirm reports from previous studies in Tanzania, that RDT introduction can lower anti-malarial drug consumption [13] and may help reduce the problem of anti-malarial drug stock-outs. This may imply that once a policy of malaria diagnostic confirmation expands to the entire country, the availability of ACT in the Tanzanian health sector might be significantly improved.

This study showed that only about a third of fever patients actually received ACT in the post RDT implementation area, despite the fact that most of the patients (94%) were seen in a facility with ACT in stock. As the use of the rapid tests increase once introduced, health workers’ performance is likely to improve since there will be an added tool in the line of care that provides more job and client satisfaction [18]. Some community studies in Tanzania and elsewhere indicate that community members are willing to receive and pay for a laboratory test prior to malaria treatment [19]. Moreover, some studies reported that clients’ demand for a malaria confirmatory test before treatment [20] and having a malaria test increased patient satisfaction with clinical care provided [18,19].

Rapid tests seemed to be performing better than microscopy. It is important to become conscious of the possibility for over-estimation of positivity rate that might result from use of RDTs. In particular, this is likely to be a problem with the use of histidine rich protein-2 based RDT devices for detecting *Plasmodium falciparum* infection, as they may continue to test positive weeks after parasite clearance. In this case, training on RDTs should stress the need for assessing other disease conditions despite a positive test for malaria, and referral to higher level of care for a microscopic examination of malaria. This is particularly important for providing quality care of malaria patient in the changing malaria transmission patterns, with a downward trend observed in many malaria endemic countries. It as well, supports present efforts to obtain accurate information about the disease burden in the population.

There are several limitations to this evaluation. First, this survey was carried out in established health facilities where both the providers and patients were aware of the presence of the study team. This may have inadvertently influenced prescription behaviour of the providers. Care was taken to use field interviewers who were local residents of the survey area. Response bias was minimized by using survey tools that recorded what was done on the survey day without longer recall periods, except for provider’s experience and training. Secondly, the survey was limited to health facilities within the HDSS areas and since the HDSS conducts many health interventions and research studies, this may render the population more health conscious than the general population. Third, the presence of RDTs in the pre-RDT implementation area and some RDT stock-outs in the post-RDT implementation area, may have underestimated the true impact of rolling out RDTs. Methodologically, a ‘before and after’ design would have provided a better measure of RDT impact in case management, but the purpose of this evaluation was to assess performance of health care system with all the bottlenecks associated with it such as stock outs of medicines, diagnostics and other medical products. In addition, prescription practices may differ from one area of practice to another due to common experiences and governing principles. This may affect the true measure of impact of RDT implementation between the study areas. Lastly, ACT stock out was another limitation that might have affected the true impact of the programme.

## Conclusions

In conclusion, the implementation of RDTs increased use of RDTs for parasitological confirmation and reduced over-treatment with ACT during high malaria transmission season in one area in Tanzania. Continued monitoring of the national RDT rollout will be needed to assess whether these changes in case management practices will be replicated in other areas and sustained over time. Additional measure such as refresher trainings, closer supportive supervisions, etc., may be needed to improve ACT targeting during low transmission seasons. The need to extend parasitological confirmation of malaria in the context of integrated community case management is becoming apparent and needs to be addressed.

## Competing interests

The authors declare that they have no competing interests.

## Authors’ contributions

IMM was responsible for the field supervision, analysis and preparation of first draft. MS and DK undertook statistical analysis and review of subsequent manuscript. BA was responsible for field supervision and review of subsequent manuscripts. RK, SPK, JS designed the study and reviewed the manuscripts. All authors have read and approved the final manuscript.

## Supplementary Material

Additional file 1: Performance of facility malaria diagnostic tests in pre- and post-RDT implementation HDSS areas. Click here for file
