# Supplementary material for: Increased use of malaria rapid diagnostic tests improves targeting of anti-malarial treatment in rural Tanzania: implications for nationwide rollout of malaria rapid diagnostic tests
Source: Malar J. 2012 Jul 2;11:221. doi: 10.1186/1475-2875-11-221 (PMC3471012; doi:10.1186/1475-2875-11-221)
Supplement: Additional file 1: — Performance of facility malaria diagnostic tests in pre- and post-RDT implementation HDSS areas. [file 1475-2875-11-221-S1.doc]

ADDITIONAL FILES

**Performance of facility malaria diagnostic tests in pre- and post-RDT implementation HDSS areas**

|  | **Malaria rapid diagnostic tests (RDT)** | | | | | | |
| --- | --- | --- | --- | --- | --- | --- | --- |
|  | **Ifakara HDSS (pre- RDT implementation)** | | | **Rufiji HDSS (post- RDT implementation)** | | | **P-value** |
|  | **n/N** | **% (95%CI)** | | **n/N** | **% (95%CI)** | |  |
| Sensitivity | 4/14 | 28.6 (10.2– 58.4) | | 58/65 | 89.2 (79.3 - 94.7) | | <0.01 |
| Specificity | 94/120 | 78.3 (70.0- 85.0) | | 210/247 | 85 (79.6 - 89.2) | | 0.51 |
| Predictive value positive | 4/30 | 13.3 (4.4- 34.0) | | 58/95 | 61.1 (48.7-72.1) | | 0.01 |
| Predictive value negative | 94/104 | 90.4 (83.0- 94.8) | | 210/217 | 96.8 (94.1-98.3) | | 0.05 |
|  | **Microscopy** | | | | | |  |
|  | **Ifakara HDSS (pre- RDT implementation)** | | | **Rufiji HDSS (post- RDT implementation)** | | | **P- value** |
|  | **n/N** | | **% (95%CI)** | **n/N** | | **% (95%CI)** |  |
| Sensitivity | 7/10 | | 70(19.1 - 95.8) | 10/13 | | 76.9 (47.9 - 92.4) | 0.18 |
| Specificity | 132/235 | | 56.2(39.6- 71.4) | 93/119 | | 78.2 (68.7 - 85.3) | 0.05 |
| Predictive value positive | 7/110 | | 6.4 (2.6-14.8) | 10/36 | | 27.8 (12.4- 51.2) | 0.49 |
| Predictive value negative | 132/135 | | 96.9 (90.8-99.0) | 93/96 | | 96.9 (90.8-99) | 0.32 |
